# Continuous subcutaneous infusion for pain control in dying patients: experiences from a tertiary palliative care center

**DOI:** 10.1186/s12904-020-00681-3

**Published:** 2020-11-10

**Authors:** Per Fürst, Staffan Lundström, Pål Klepstad, Peter Strang

**Affiliations:** 1 Department of Oncology Pathology, Karolinska Institutet, Stockholm, Sweden; 2 Palliative Medicine, Stockholms Sjukhem Foundation, Stockholm, Sweden; 3 Department of Circulation and Medical Imaging, Faculty of Medicine, Norwegian University of Science and Technology, Trondheim, Norway; 4 European Palliative Research Centre, Department of Clinical and Molecular Medicine, Faculty of Medicine, Norwegian University of Science and Technology, Trondheim, Norway; 5 Department of Anesthesiology and Intensive Care medicine, St. Olavs Hospital, Trondheim University Hospital, Trondheim, Norway

**Keywords:** Cancer, Methadone, Opioids, Pain, Subcutaneous, Infusion

## Abstract

**Background:**

Continuous subcutaneous infusion (CSCI) via ambulatory infusion pump (AIP) is a valuable method of pain control in palliative care. When using CSCI, low-dose methadone as add-on to other opioids might be an option in complex pain situations. This study aimed to investigate the effects, and adverse effects, of CSCI for pain control in dying patients, with particular interest in methadone use.

**Methods:**

This was an observational cohort study. Imminently dying patients with pain, admitted to specialized palliative inpatient wards and introduced on CSCI, were monitored daily by staff for symptoms (Integrated Palliative Care Outcome Scale - IPOS), sedation (Richmond Agitation and Sedation Scale – RASS), performance status (Eastern Cooperative Oncology Group - ECOG) and delirium (Confusion Assessment Method - CAM).

**Results:**

Ninety-three patients with a median survival of 4 days were included. Of the 47 patients who survived ≥3 days, the proportion of patients with severe/overwhelming pain decreased from 45 to 19% (*p* < 0.001) after starting CSCI, with only a moderate increase in morphine equivalent daily dose of opioids (MEDD). Alertness was marginally decreased (1 point on the 10-point RASS scale, *p* = 0.001), whereas performance status and prevalence of delirium, regardless of age, remained unchanged.

Both patients with methadone as add-on (MET, *n* = 13) and patients with only other opioids (NMET, *n* = 34), improved in pain control (*p* < 0.05 and 0.001, respectively), despite that MET patients had higher pain scores at baseline (*p* < 0.05) and were on a higher MEDD (240 mg vs.133 mg). No serious adverse effects demanding treatment stop were reported.

**Conclusions:**

CSCI via AIP is an effective way to reduce pain in dying patients without increased adverse effects. Add-on methadone may be beneficial in patients with severe complex pain.

## Background

Pain is a common clinical problem in palliative care [1]. Eighty percent of patients with advanced cancer and 67% of patients with cardiovascular disease or chronic obstructive pulmonary disease will experience moderate to severe pain at the end of their lives [2]. A better understanding of pain mechanisms has improved pain therapy in recent years [3]. Especially, cancer patients in palliative care are adequately treated with low or moderate doses of opioids and only about 10 % need daily morphine doses over 600 mg [4]. However, with progressive deterioration at the end of life, continuous subcutaneous infusions (CSCI) become increasingly important to continue pain control in the dying [5]. The use of an ambulatory infusion pump (AIP), a small, portable pump that delivers medication via a thin subcutaneous catheter, has several advantages for pain control: It ensures a steady infusion of drugs, with reliable absorption if inserted in unaffected tissue, and allows combinations of drugs to be administered parenterally in a manner that is more convenient than repeated and painful injections. Thus, there is no need for an intravenous access [6, 7]. However, despite encouraging data in palliative care in earlier phases, there is a lack of prospective studies on the use of CSCI in the imminently dying.

Low-dose add-on peroral methadone in combination with other opioids for pain is proposed to be a useful alternative to methadone therapy for better pain control at the end of life [8–12]. The addition of methadone is reported to improve pain relief in complex pain situations but, so far, only peroral or intermittent parenteral administration of low-dose add-on methadone has been studied, routes that are often not feasible in the imminently dying patient [13–15]. Alternative routes of methadone administration are therefore needed and there is a need for further exploration of the effects, and possible adverse effects, of CSCI in this patient group [13, 14].

Whereas patient-reported outcomes are encouraged, this is not possible in imminently dying patients, as the majority of them are not able to complete a questionnaire for obvious reasons, such as, terminal delirium, extreme tiredness or lowered level of consciousness. For this reason, very few studies on symptoms and symptom control have been performed in the very last days of life, a period which is important both for the patient and for their families. A way to overcome this problem is to use validated instruments allowing proxy measures, e.g. assessments made by staff.

The primary aim of the study was to report the effects on pain intensity and occurrence of adverse effects, e.g. sedation, confusion, and respiratory depression, when prescribing an AIP for CSCI in imminently dying patients. A secondary aim was to specifically study the effects of the addition of low-dose methadone to a CSCI comprising another opioid in this patient group.

## Methods

The methods and results sections are reported, when applicable, based on the Strengthening the Reporting of Observational Studies in Epidemiology (STROBE) criteria [16].

### Study design

At the specialized palliative care in-patient unit at Stockholm Sjukhem Foundation, we conducted an observational cohort study in patients who were prescribed CSCI via AIP for symptom management during the last days of life. Patients were included from February 1, 2019 to January 22, 2020 and followed up daily, with the last follow-up on January 28, 2020.

### Population

All Swedish-speaking patients over the age of 18 years who were neither sedated nor unconscious and who, during daytime (7 am – 8 pm) as part of their regular care, were prescribed a CSCI of drugs were asked to participate in the study. Inclusions were not possible during other hours due to limited available resources for performing the inclusion procedure.

### Definition of total cohort and main study group

The total cohort consisted of all patients that were included. The main study group consisted of those patients from the main study group, who had pain at inclusion and who survived for three days or more.

### Variables

For every patient, baseline data on age, sex, indication for CSCI, prevalence, intensity and type of pain were registered. Daily registrations covered the previous 24 h and included medications, site of SC infusion, skin irritation around the SC needle, and other symptoms including patient anxiety, alertness, performance status and occurrence of confusion.

### Data sources and rating scales

As the patients were in an acute dying phase with rapid deterioration from day to day, the study applied exclusively instruments allowing proxy measures (staff measures). The daily assessments were performed by the registered nurse responsible for the patients’ care on that day and could thus be performed by various individuals from day to day. The proxy version of the Integrated Palliative Care Outcome Scale (IPOS) was used to assess the patient’s symptoms and relatives’ concerns [17]. The IPOS-variables pain and anxiety were reported on an ordinal scale estimating how much the patient was affected, ranging from 0 (not at all) to 4 (overwhelmingly/worst pain imaginable) and these numerical estimates were then used to calculate the pain and anxiety levels. The Richmond Agitation and Sedation Scale (RASS) was used to indicate the level of patient alertness (+ 4 combative via 0 alert and calm to − 5 unarousable) [18, 19]. Performance status was assessed according to the Eastern Cooperative Oncology Group (ECOG/WHO) performance status (0 fully active to 5 dead) and the Confusion Assessment Method (CAM) instrument was used for assessing delirium in a Yes/No format [20, 21].

### Study size

This is a descriptive study, and no sample size calculation was performed.

### Statistical methods and missing data

Descriptive statistics are presented with both means and medians as appropriate. T-tests were used to compare age and survival, and, for other variables, the following non-parametric tests were applied: chi-square test to compare proportions, Mann–Whitney U test to compare independent groups and Wilcoxon signed-rank test to compare dependent groups.

From initiation of CSCI until day 3, there were missing data on an average of 5 patients per day of the total study cohort, though in no case for more than one consecutive day. For missing data, the last observation carried forward was used. Analysis was performed using SPSS Version 26.

Ethical approval (2018/2103–31/1) was obtained from the Regional Ethical Review Board (Stockholm, Sweden) and all participants consented to inclusion in the study.

## Results

**Fig. 1:**
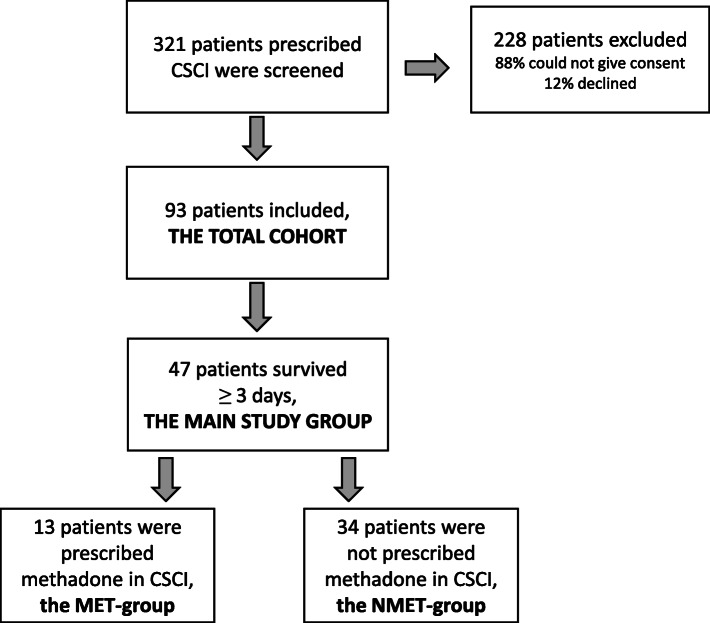
Flow chart of patient selection

During the study period, 321 patients were prescribed CSCI and a total of 93 were included in the study intended to be followed prospectively. Of those not included, 88% were assessed as too ill to give formal consent and 12% abstained (Fig. 1). Mean age of the included patients was 76.3 years (median 77 years), 57% were women, (Table 1). Indications for CSCI were: inability to swallow due to deterioration of general condition in 49 (53%) patients, uncontrolled pain with oral pain medication in 27 (29%) patients, bowel obstruction in 6 (6%) patients and unspecified reasons in 11 (12%) patients. On day 0 (initiation of CSCI), the mean pain score was 2.2 (median 2), with 5 (5%) patients rating no pain (Table 2). Two thirds of the reported pain mechanisms were combined nociceptive and neuropathic. For other characteristics, see Table 1.

**Table 1:** Patient characteristics and pain mechanisms on the day of CSCI initiation

	*Total cohort*	*Main study group,* ≥ *3 days of survival*		
		*Total*	*Methadone in CSCI*	*No methadone in CSCI*
	*n* = 93	*n* = 47	*n = 13*	*n = 34*
**Females** *n* (%)	53 (57)	25 (53)	5 (38)	19 (56)
**Age** *years*				
mean (SD)	76.3 (10.6)	75.6 (12.1)	68.7 (11.7)	78.2 (11.5)*
median (IQR)	77 (12)	76 (16)	71 (14.5)	77.5 (16.3)
**Survival** *days*				
mean (SD)^a^	8 (10)	9 (9)	13 (8)	8 (10)*
median (IQR)	4 (5)	5 (9)	14 (14)	4 (5)
**Midazolam**				
used midazolam *n (%)*	63 (68)	30 (64)	7 (54)	23 (68)
Dose, mg/24 h				
mean (SD)	8.0 (11)	7.1 (9)	18.8 (11.2)	8.7 (7.4)
median (IQR)	5 (10)	5 (10)	20 (25)	6.3 (5)
range (mg)	0–50	0–37.5	2.5–31	2.5–37.5
**Performance status**				
mean (SD)	3.4 (0.9)	3.3 (1)	3.4 (0.9)	3.5 (0.5)
median (IQR)	4 (1)	4 (IQR 1)	4 (1)	4 (1)
**Reported pain mechanism** *n (%)*	71 (76)	37 (80)	10 (77)	25 (53)
Mixed nociceptive and neuropathic pain	47 (66)	26 (70)	6 (60)	18 (72)
Nociceptive pain	19 (27)	6 (16)	2 (20)	4 (16)
Neuropathic pain	5 (7)	5 (14)	2 (20)	3 (12)
**Malignant diagnoses** *n (%)*				
Gastrointestinal (other than pancreas)	25 (27)	12 (26)	3 (23)	9 (26)
Lung	19 (21)	11 (23)	5 (38)	6 (18)
Urogenital (other than prostate)	11 (12)	6 (13)	2 (15)	4 (12)
Pancreas	7 (8)	2 (4)		2 (6)
Breast	7 (8)	3 (6)		3 (9)
Hematological	5 (5)	3 (6)	1 (8)	2 (6)
Prostate	2 (2)	1 (2)		1 (3)
Head and neck	2 (2)	1 (2)		1 (3)
Skin	1 (1)	1 (2)	1 (8)	
Other/unknown origin	6 (6)	3 (6)	1 (8)	2 (6)
**Non-malignant diagnoses** *n (%)*				
lung fibrosis	2 (2)	2 (4)		2 (6)
COPD	2 (2)			
Heart failure	2 (2)			
Renal failure	1 (1)	1 (2)		1 (3)
Stroke	1 (1)	1 (2)		1 (3)

^a^After exclusion of one outlier with 125 days of survival

Significance of difference between the subgroups of the main study group that were prescribed methadone in CSCI (*n* = 13) and those who were not (*n* = 34):*p < 0.05

This table shows the basic characteristics of the groups analyzed in the study on the day when the continuous subcutaneous infusion was started. The total cohort was all 93 patients who received continuous subcutaneous infusion (CSCI) and were included. The main study group consisted of the 47 patients who survived at least 3 days. Of these, 13 patients were prescribed methadone in CSCI and 34 were not.

(*n* number of patients, *SD* standard deviation, *IQR* inter quartile range)

**Table 2:** Pain and opioid doses

	*Total cohort*	*Main study group,* ≥ *3 days of survival*					
		*Total*		*Methadone in CSCI*		*No methadone in CSCI*	
	*n* = 93	*n* = 47 (13 + 34)		*n = 13*		*n* = 34	
	*Day 0*	*Day 0*	*Day 3*	*Day 0*	*Day 3*	*Day 0*	*Day 3*
**Level of pain** *(0–4)*^*a*^							
Mean (SD)	2.2 (1.1)	2.2 (1.2)	1.5 (1.2)***	2.9 (1.0)	2.1 (1.3)*	2.0 (1.1)	1.2 (1.1)***
Median (IQR)	2 (2)	2 (2)	2 (2)	3 (1)	2 (2)	2 (2)	1 (1)
Severe to overwhelming pain (scores 3–4) *n (%)*	39 (42)	21 (45)	9 (19)***	10 (77)	6 (46)*	11 (32)	3 (9)***
**MEDD**^b^ **of opioids,** *mg*							
Mean (SD)	179 (175)	184 (181)	205 (182)*	306 (257)	354 (225)	133 (108)	142 (116)*
Median (IQR)	120 (169)	123 (151)	150 (210)	240 (310)	300 (193)	113 (120)	105 (125)
Range	22.5–1020	22.5–1020	30–870	22.5–1020	75–870	30–435	30–563
**Methadone,** *mg*							
Mean (SD)	5.5 (4.6)	–	–	7.5 (4.8)	7.7 (4.5)	–	–
Median (IQR)	5 (7.5)	–	–	5 (5)	5 (5)	–	–
Range	0–20	–	–	2.5–20	5–20	–	–

^a^Ordinal scale from 0 (not at all affected by pain) to 4 (overwhelmingly)

^b^Total morphine equivalent (oral) daily doses

Significance of difference from day 0 to day 3:*p < 0.05; ** *p* < 0.01; ***p ≤ 0.001. The comparisons are made for the main study group (*n* = 47), for the MET group (*n* = 13) and the NMET group (*n* = 34), respectively

The table refers partly to the situation at the day for start of the continuous subcutaneous infusion (CSCI), and partly 3 days later. It is divided into the different groups analyzed in the study, i.e. the total cohort which is all 93 patients who received CSCI from day 0 and the 47 patients who survived at least 3 days and who make up the main study group. The 13 patients in the main study group who were prescribed methadone in CSCI are the MET group and the 34 who were not prescribed methadone are the NMET group. The table shows levels of pain at the different times, and also the doses of opioids that the patients were prescribed, including methadone.

(*n* number of patients, *SD* standard deviation, *IQR* inter quartile range)

Three patients, one of whom received methadone, experienced local erythema which disappeared within one day after changing the injection site. In all three cases, the needle had been in place for at least 5 days.

### Patients with 3 days or more of survival time

To enable the study of the effects and adverse effects of opioids over time, patients with no pain and with a survival of less than 3 days, as well as patients who also received additional palliative sedation, were excluded from further analyses, leaving 47 patients in the main study group. This group included patients receiving midazolam prescribed for anxiety, but not for sedation. The median survival in this cohort was 5 days (mean 9). For further characteristics, see Table 1.

#### Adjuvant analgesics and opioids

Thirty-three patients (70%) used peroral non-opioids and/or adjuvant analgesics on the day of initiation of CSCI (mean 1.7 adjuvants), including corticosteroids, paracetamol (acetaminophen), NSAIDs, antidepressants and gabapentinoids. The following opioids (oral or parenteral) were used in the main study group until the switch to CSCI: Oxycodone in 61% of the cases, morphine in 23%, fentanyl patches in 14% and hydromorphone in 2% of the cases. All oral opioids were discontinued upon conversion to CSCI. The total mean morphine equivalent daily dose (MEDD) via CSCI at baseline was 184 mg. In eight cases, only opioids were used in the AIP, in 17 cases the opioids were combined with one additional drug, in 16 cases with two others and in six cases with three other drugs. For specific details on CSCIs containing methadone, see Table 3.

**Table 3:** Continuous Subcutaneous Infusions for the MET group

*Age-group*	*Patient*	*Malignancy*	*Max Methadone dose/24 h. in CSCI (mg)*	*Local toxicity*	*Regular opioid in CSCI*	*Other drugs in CSCI*
40–49	1	Liver	20	no	morphine	midazolam, haloperidol
	4	Thyroid	10	no	hydromorphone	midazolam, hyoscine butylbromide, metoclopramide
60–69	2	Abdominal	5	no	oxycodone	hyoscine butylbromide
	6	Lung	5	no	oxycodone	midazolam, hyoscine butylbromide, haloperidol
	7	Lung	10	no	hydromorphone	midazolam, metoclopramide
	13	Lung	5	no	hydromorphone	midazolam, hyoscine butylbromide, haloperidol
70–79	5	B-cell lymfoma	7.5	yes	oxycodone	midazolam
	8	Bladder	5	no	oxycodone	
	9	Bladder	20	no	hydromorphone	midazolam, haloperidol
	10	Lung	10	no	hydromorphone	midazolam, haloperidol
	11	Lung	5	no	hydromorphone	midazolam, haloperidol
80–89	3	Colon	5	no	oxycodone	
	12	Merkel cell	10	no	oxycodone	midazolam, haloperidol

This table describes the characteristics of the 13 patients who were prescribed methadone in continuous subcutaneous infusion (CSCI), the MET group. By local toxicity is meant whether or not skin erythema occurred.

#### Pain and other symptoms from day 0 to day 3

##### Pain

The proportion of patients severely or overwhelmingly affected by pain decreased from 45 to 19% (*p* < 0.001) and the mean pain score from 2.2 (median 2) to 1.5 (median 2) on the five-point pain IPOS scale, (Table 2).

##### Alertness

The mean level of alertness went from − 0.2 to − 1.2, i.e. 1 point on a 10-point scale (*p* = 0.001), (Table 4). During the same period, doses of midazolam (median 7.5 to 9.4 mg, *p* = 0.31) and performance status did not change significantly.

**Table 4:** Adverse effects

	*Total cohort*	*Main study group,* ≥ *3 days of survival*					
		*Total*		*Methadone in CSCI*		*No methadone in CSCI*	
	*n = 93*	*n = 47*		*n = 13*		*n = 34*	
	*Day 0*	*Day 0*	*Day 3*	*Day 0*	*Day 3*	*Day 0*	*Day 3*
**Alertness**^a^							
Mean (SD)	−0.5 (1.2)	−0.2 (1)	−1.2 (1,7)***	0.4 (0.9)	−0.9 (1.7)*	− 0.4 (1)	−1.3 (1.7)*
Median (IQR)	0 (1)	0 (2)	−1 (2)	0 (1)	0 (2)	−0.5 (1)	−1 (2)
**Delirium**^b^							
Prevalence *n (%)*	27 (29)	14 (30)	15 (32)	4 (31)	5 (39)	10 (29)	10 (29)
Prev. < 75 years	12 (32)	7 (33)	9 (43)	4 (31)	5 (39)	8 (32)	7 (28)
Prev. ≥ 75 years	15 (27)	7 (27)	6 (23)	3 (30)	2 (20)	12 (27)	4 (9)
**Anxiety** ^c^							
median (IQR)	3 (1)	3 (1)	2 (2)	3 (1)	2 (1.8)	3 (1.5)	2 (2)

^a^Ordinal scale from + 4 (combative) to −5 (unarousable)

^b^Prevalence of confusion/delirium (Yes/No)

^c^Ordinal scale from 0 (not at all) to 4 (always)

Significance of differences from day 0 to day 3:*p < 0.05; ***p ≤ 0.001

In this table, the status of all 93 patients regarding alertness, prevalence of delirium and anxiety on the day of initiation, day 0, of continuous subcutaneous infusion (CSCI) is reported under total cohort. The main study group is the 47 patients who survived at least 3 days. The changes from day 0 to day 3 in adverse effects is shown for the total group and also for the 13 patients who received methadone in CSCI and the 34 patients who did not.

(*n* number of patients, *SD* standard deviation, *IQR* inter quartile range)

##### Delirium

Delirium was seen in 30% of the patients at day 0 and did not change significantly over time (day 0 vs. day 3). Neither were there differences between patients under or above 75 years of age, (Table 4).

##### Anxiety

At baseline, 52% of the patients were reported to be anxious most of the time or always. On day 3 however, this proportion was significantly lower, 26% (*p* = 0.04), (Table 4).

### Patients receiving methadone

Thirteen patients received low-dose methadone added to the continuous infusion (MET). The median time of survival from initiation of CSCI was 14 days for the MET group compared with 4 days for the group of patients whose CSCIs contained no methadone (NMET), respectively (*p* = 0.044), (Table 1). Of the 13 patients in the MET group, 10 were prescribed methadone for the first time on day 0 and the other 3 were previously on peroral methadone due to previous, insufficient pain control. After 3 days, the dose was unchanged in 72%, increased in 18% and decreased in 10% of the cases. The most common starting dose of SC methadone was 5 mg per 24 h (55%), (Table 2). All the patients used methadone until the CSCI was ended, in all cases due to death.

#### Opioids

At baseline, the MEDD of opioids via CSCI for MET (median 240 mg) was almost twice the dose for NMET (median 133 mg), (*p* = 0.004). From days 0 to 3 there was an increase in opioid doses for NMET (*p* = 0.02).

#### Pain and other symptoms from day 0 to day 3

##### Pain

On day 0, MET compared with NMET patients had more severe pain (*p* = 0.02) and a higher proportion of severe/overwhelming pain, 77% vs. 32% (*p* = 0.009), (Table 2). On day 3, pain scores were significantly reduced for both MET and NMET patients (*p* = 0.04 and *p* = 0.001, respectively) and the proportion of severe pain was lower, (Table 2).

##### Alertness

MET started at a slightly higher alertness level than NMET (*p* = 0.02). The levels dropped from 0.4 to − 0.9 (median 0 to 0; *p* = 0.02) and from − 0.4 to − 1.3 (median − 0.5 to − 1; *p* = 0.02), respectively, (Table 4).

There were no significant differences between MET and NMET over time regarding doses of midazolam (*p* = 0.054), performance status, anxiety, or prevalence of delirium, (Table 1 and Table 4).

#### Serious adverse effects

One MET patient had a respiratory ratio lower than eight breaths per minute on days three and four. No interventions were needed, and from day five he had normal respiratory ratios until death on day nine.

## Discussion

In this study on symptom relief during the very last days of life, we report that the use of AIP for CSCI for pain control in dying patients contributed to improved analgesia with no clinically significant change in adverse effects. In addition, CSCI with low-dose methadone in combination with other regular opioids was prescribed at the individual physician’s discretion and resulted in improved analgesia for patients with the most severe pain.

Several studies and guidelines report on the treatment of severe pain in patients in palliative care in less advanced stages [3, 22–24]. However, few studies investigate pain and symptom management in the imminently dying, due to the well-known problems related to symptom assessment [25, 26]. The short median survival time of four days confirms that the patients in this study were at the very end of life. In this setting, the most common reason for initiating CSCI was general deterioration causing impaired oral intake. Another major reason was to provide better pain relief by converting to parenteral drug delivery and, when judged necessary, by adding parenteral methadone. The need for better pain relief may be due to mixed nociceptive and neuropathic pain in two thirds of the patients in this study, a combination of pain mechanisms often difficult to treat [14].

Significant reduction in pain was seen for the entire group of patients who received CSCI with opioids for pain and who could be followed for at least three days. This was regardless of whether the pain was measured as the proportion of patients with severe pain or as the median and average pain scores based on the Likert scale for pain in the IPOS [17]. Advantages in CSCI administration of opioids in relation to pain control include a more stable serum concentration of the drug with avoidance of end-of-dose interval breakthrough pain and less adverse effects occurring at high peak concentrations following intermittent injections which allows a more adequate opioid dose titration [6, 27, 28]. In the imminently dying patients a rotation from oral intake to parenteral routes might be especially beneficial, as the oral route, including swallowing and absorption, might be unreliable in the last days and hours of life.

In this study, it was not recorded whether the same opioid was used when switching to CSCI or whether an alternative opioid was used. There may exist a cross-tolerance between opioids, meaning that a different opioid has a better analgesic effect than expected from equianalgesic tables [29]. However, a review by Schuster et al. 2018 confirmed the stated findings in the Cochrane review from 2004, that although widely practiced, robust evidence for the benefit of opioid rotation is still lacking [30, 31].

Patients who required the addition of methadone for analgesia had higher pain scores and opioid doses at initiation of CSCI. Thus, the use the co-prescription of low-dose methadone with another opioid for CSCI via AIP was mainly initiated in patients with complex pain of high intensity, in order to improve pain control without large dose escalations of the regular opioid. This is in line with the study by Mercadante et al., that described how addition of methadone to another opioid in patients with cancer pain may reduce the need for opioid dose escalation [32]. As shown in this study, the apparent beneficial analgesic effect adding of low-dose methadone to another opioid for administration by CSCI is promising and reflects observations reported for oral administration [8–12].

There was a statistically significant decrease in alertness (*p* < 0.001). However, the clinical impact is probably minor as the change was 1.0 in a 10-grade scale, i.e. a 10 % deterioration, in acutely dying patients. Besides this, there were neither any increase in intensity of other adverse symptoms nor any serious adverse effects that demanded specific interventions. Moreover, we observed no significant differences over time or between MET and NMET regarding performance status, anxiety levels or doses of the anxiolytic midazolam. These observations agree with the general observation that adverse effects such as sedation, constipation, and respiratory depression are associated with the pharmacology of opioids as a class, and similar reactions are expected regardless of route [28].

Notably, those in the subgroup MET had higher total opioid doses, still they survived marginally longer. The MET group’s longer survival from introduction to death may possibly be explained by their more complex pain situation, possibly resulting in an earlier introduction of CSCI.

Particularly interesting is that the prevalence of delirium in the main study group did not change over time and did not differ between patients younger or older than 75 years of age. To the best of our knowledge, differences in adverse effects following introduction of CSCI for pain in different age groups has not been described before, but our findings are consistent with previous studies indicating that a steady infusion of subcutaneous drugs may be better tolerated [6, 33].

Problems with local skin irritation at the site of CSCI has been associated with methadone but are reported to resolve with site rotation [15, 34–36]. In our prospective study, only low-doses of methadone were infused and we found only three cases of skin irritation, two in the NMET group and one in the MET group. The needle had been in the same place for at least five days which, therefore, may be as likely an explanation for the dermal erythema as the drugs in the CSCI. We therefore suggest that the risk of skin irritation should not be considered a major limiting factor for the use of methadone in CSCI.

There was a significant difference in mean age between the MET group, 68 years, and the NMET group, 78 years, (*p* < 0.05). We described a similar difference in a study from 2020 on a different sample [13]. To the best of our knowledge this has not been studied before and we do not know the underlying reason.

We do recognize some limitations of the study. First, as accounted for in the methods section, all patients receiving a CSCI were not included, usually due to illness and of ability to provide informed consent, as well as some patients refraining from participation. Second, as methadone, according to clinical guidelines, was mainly prescribed to patients with the most severe pain, the MET and NMET groups differed significantly at baseline as regards pain intensity and MEDD. Still, such comparisons of subgroups are of interest, as they reflect a clinical reality. Comparable control groups are difficult to achieve in a population of dying patients in need of parenteral drug administration, and randomization with control groups is seldom possible, for ethical reasons. Third, due to the severe illnesses of the patients, observer ratings had to be used. By definition, observer assessment may not directly reflect the patient’s experiences, meanwhile, they allow studies in the last days and hours of life, on an important patient group that is often excluded from studies. The assessments were based on subjective judgements by the registered nurses who performed the registrations. Consequently, the assessments came to a certain extent to depend on each individual’s level of knowledge, skills, and personal attitudes. Finally, this is a single-center study reflecting the experiences at one particular setting.

Strengths include the prospective design until death and the use of validated instruments for symptom assessments. Another strength is that the proxy assessments were performed by trained personnel, which may have contributed to improved assessments of adverse effects [37].

## Conclusions

Regardless of age, CSCI via AIP for pain seems effective in reducing pain in dying patients without any substantial increase of adverse effects such as delirium or respiratory depression. An addition of low-dose methadone may be beneficial for CSCIs for patients with severe cancer pain at the end of life. The effectiveness of low-dose methadone in combination with the patient’s regular opioid needs further investigation, preferably with a randomized controlled trial.

## Data Availability

The datasets used and/or analyzed during the current study are available from the corresponding author on reasonable request.

## Abbreviations

CSCI: Continuous subcutaneous infusion
AIP: Ambulatory infusion pump
CAM: Confusion Assessment Method
ECOG: Eastern Cooperative Oncology Group
hrs.: Hours
IPOS: Integrated Palliative Care Outcome Scale
IQR: Inter quartile range
MEDD: Morphine equivalent daily dose of opioids
MET: Patients with methadone as add-on
mg: Milligrams
n: Number
NMET: Patients on opioids other than methadone only
NSAID: Non-steroidal anti-inflammatory drug
RASS: Richmond Agitation and Sedation Scale
SC: Subcutaneous
SD: Standard deviation
STROBE: Strengthening the Reporting of Observational Studies in Epidemiology
